# A Unique Case of Cellulitis Secondary to Mycobacterium chelonae in a Patient With Monoclonal Gammopathy of Undetermined Significance

**DOI:** 10.7759/cureus.57514

**Published:** 2024-04-03

**Authors:** Stevan Oluic, Mohamed Hassan, Mohamad El Labban, Hussein Guleid, Waclaw Wedzina

**Affiliations:** 1 Internal Medicine, Mayo Clinic Health System, Mankato, USA; 2 Hospital Medicine, Mayo Clinic Health System, Mankato, USA

**Keywords:** cutaneous tuberculosis and atypical mycobacterial infections, skin and soft tissue, monoclonal gammopathy of undetermined significance (mgus), non-tuberculosis mycobacteria, mycobacterium chelonae

## Abstract

We report a case of an 84-year-old patient with Monoclonal Gammopathy of Undetermined Significance (MGUS) treated with multiple courses of antibiotics and steroids before being diagnosed with *Mycobacterium chelonae* infection. It is known that MGUS affects both humoral and cellular immunity with impairment of antibody production, function of T-cells, natural killer (NK) cells, and dendritic cells. This case report demonstrates the need to consider patients with MGUS as immunocompromised and draws attention to the correlation between MGUS and *Mycobacterium *infections. The delay in diagnosis exemplifies the importance of considering atypical pathogens and involving sub-specialists early in the treatment of infections in patients with a history of MGUS.

## Introduction

Non-tuberculosis mycobacteria (NTM) have been historically seen as harmless colonizers or contaminants recovered from water, animals, milk, and soil surfaces. Advancements in diagnostic medicine, such as gene sequencing and polymerase chain reaction-restriction fragment length polymorphism (PCR-RFLP) analysis, have increased the detection and implication of NTM in pathogenic diseases. Runyon was the first to classify NTM into two groups: rapidly growing NTM and slowly growing NTM [1]. Progressive pulmonary disease, superficial lymphadenitis, disseminated disease, and skin and soft tissue infections are the four clinical syndromes associated with NTM infections [2]. The disseminated disease usually occurs in immunocompromised individuals, such as those with a Human Immunodeficiency Virus (HIV) infection [2]. The prevalence of NTM in the United States has been increasing over the past decade (6.8/100,000 in 2008, 11.7/100,000 in 2015) [3]. *Mycobacteria chelonae* (MC) is a rapidly growing NTM associated with skin and soft tissue infections [4]. Presenting symptoms can include cutaneous nodules with purplish discoloration, relapsing abscesses, and chronic discharging sinuses [5]. A retrospective study of 63 patients with NTM-related skin and soft tissue infections showed that MC infection is likely to present as multiple lesions [6].

Monoclonal gammopathy of undetermined significance (MGUS) is a clinical syndrome characterized by an abnormal presence of monoclonal immunoglobulins and/or an abnormal ratio of free immunoglobulin light chains in the blood and/or urine. Although the mechanism is not clear, several studies observed an increase in infections in patients with MGUS [7-9]. A study of 5,326 patients with MGUS found that they had twice the risk of developing any infection [9]. More specifically, Bida et al. found that MGUS diagnosis was associated with a 9.1-fold increase in the relative risk of mycobacterial infections [8]. We present a unique case of cellulitis secondary to *Mycobacteria chelonae* in a patient with MGUS. 

## Case presentation

We report a case of an 84-year-old woman with a past medical history significant for well-controlled hypertension, hypertrophic cardiomyopathy, chronic kidney disease stage 3a, secondary polycythemia, and MGUS. She presented to the internal medicine outpatient clinic for persistent swelling and redness of the second and third right metacarpophalangeal joints. She had a transthoracic echocardiogram one month prior to the presentation, after which she developed redness, warmth, and tenderness in the right antecubital fossa, where a peripheral IV line was placed during her echocardiogram. The swelling progressed to involve the dorsal side of her right hand. She was initially seen in urgent care and no laboratory tests or imaging was obtained at that time. She had no fever, chills, or other systemic signs of the infection. She was diagnosed with right-hand cellulitis and placed on Doxycycline 100 mg twice daily for seven days. Mupirocin, 2% ointment, was prescribed. Her symptoms improved initially, but they became progressively worse after stopping antibiotics. She presented to our clinic 18 days after her initial visit to urgent care. She remained afebrile with no systemic signs of infection. White blood cells and C-reactive protein were normal (Table 1, Column A).

**Table 1:** Laboratory results

	A	B	C
White blood cells ( 3.4-9.6 x 10^9^/L )	6.8x10^9^/L	7.4 x10^9^/L	
C-reactive protein ( < 5.0 mg/L)	<3 mg/L		5.2 mg/L
Erythrocyte sedimentation rate (0-29 mm/1 h)		1 mm/1 h	
Uric Acid ( 2.7-6.1 mg/dl)			7.5 mg/dl

**Figure 1:**
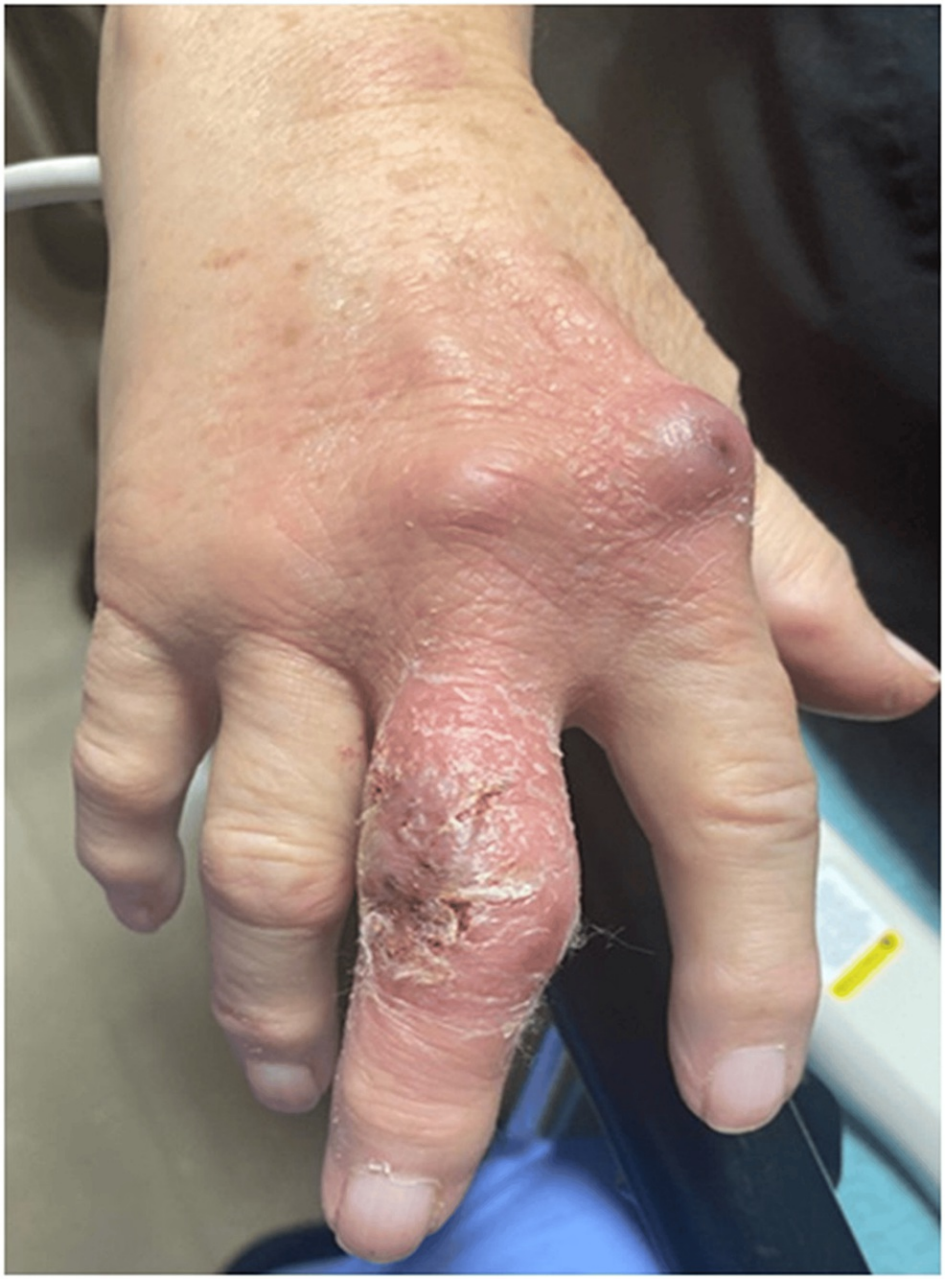
Open wound over the dorsal aspect of the third right digit

Due to concerns for the failure of oral antibiotics and worsening cellulitis, she was sent to the emergency department (ED) for possible initiation of IV antibiotics. In the ED, a bedside ultrasound was performed, which showed subcutaneous fluid and joint effusion in the right second and third metacarpophalangeal joints, which was presumed to be reactive due to ongoing cellulitis. The patient was started on cefdinir 300 mg twice daily for seven days and discharged home with instructions for outpatient follow-up. She returned to the clinic upon completion of antibiotics with little to no improvement. It was surmised that she failed two different oral antibiotic regimens and was sent back to the ED with a request to consider hospitalization and consultation by a hand surgeon and infectious disease specialist. Repeat laboratory values were within the normal range (Table 1, Column B). An X-ray did not demonstrate osseous abnormalities. The decision was made to trial corticosteroids and the patient was discharged from the ED on a 10-day course of prednisone 20 mg daily. She returned to the clinic after five days and had a complete resolution of her symptoms. However, at a 10-day follow-up, she experienced a return of redness and swelling with no systemic signs of infection. She was also started on allopurinol 50 mg daily, given mildly elevated C-reactive protein and uric acid levels (Table 1, Column C). After a couple of days, the redness and swelling significantly worsened, and she developed an open wound over the dorsal aspect of the third right digit with purulent drainage (Figure 1).

**Figure 2:**
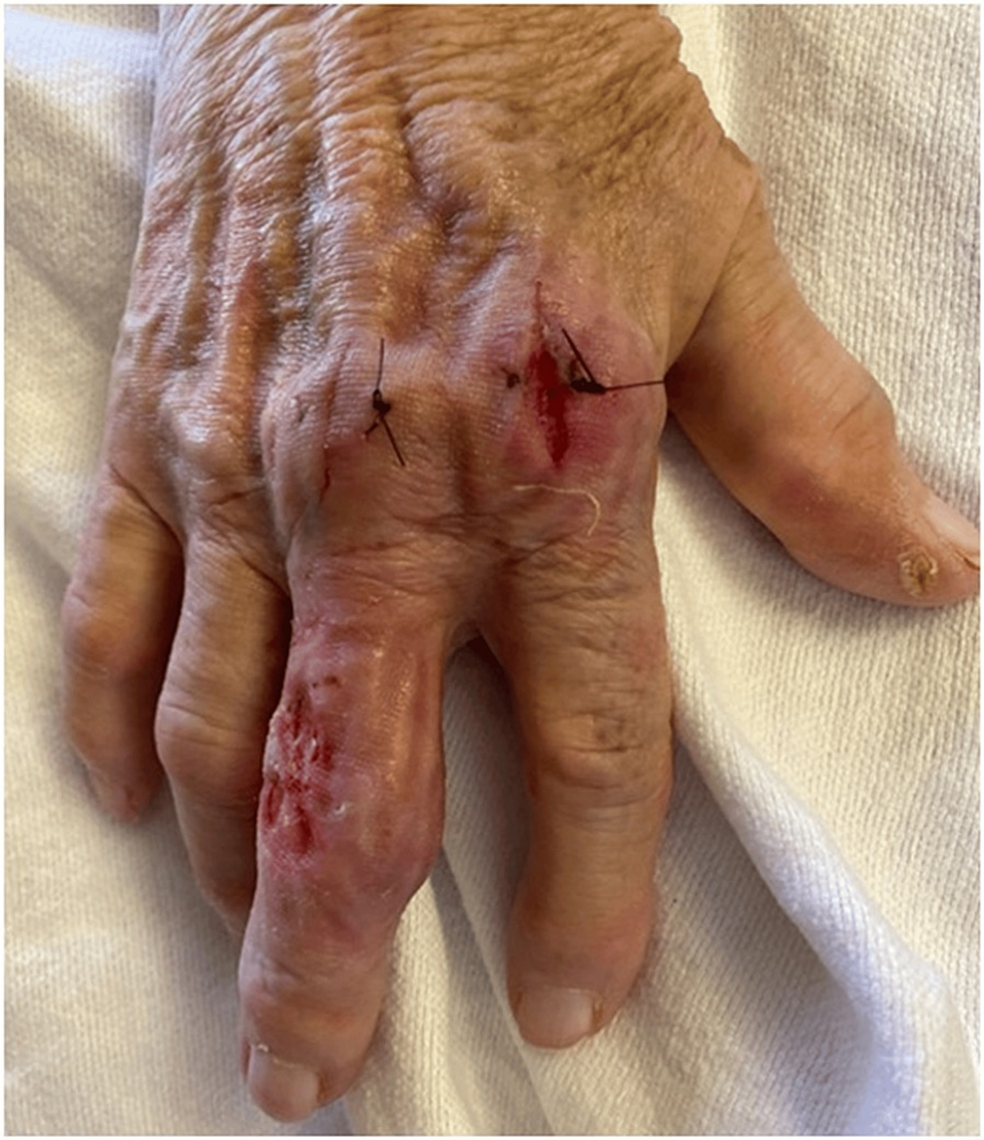
Wound after incision and drainage

She was admitted to the hospital, and a hand surgeon performed incision and drainage (Figure 2). There was an immediate rush of purulent fluid. The joint capsule was not affected. Cultures were taken, and she was started on cefazolin. Infectious disease (ID) service was consulted, and cefazolin was switched to ceftriaxone and vancomycin until intraoperative cultures were available.

Blood cultures taken during the hospitalization showed no growth. The fluid from the finger sent for analysis showed a few white blood cells and no organisms. Cultures taken intraoperatively were negative for fungal growth but came back positive for *Mycobacterium chelonae*. Based on the antibiogram susceptibility, she was started on a four-month course with azithromycin and linezolid.

## Discussion

Monoclonal Gammopathy of Undetermined Significance is considered an early stage of multiple myeloma (MM), where the production of monoclonal protein is present without systemic effects. It is estimated that 1% of MGUS progress to MM yearly [10]. Patients with MGUS also have a higher rate of renal disease, ischemic heart disease, peripheral neuropathy, and infections compared to those without MGUS [11]. A two to three-fold increase in the relative risk for viral and bacterial infections has been documented in patients with MGUS [11]. It is known that both humoral and cellular immunity are affected [11], with impairment of antibody production, function of T-cells, natural killer (NK) cells, and dendritic cells [10,11]. This immune dysregulation likely made our patient susceptible to prolonged skin infection with an uncommon pathogen.

*Mycobacterium chelone* is an opportunistic pathogen known for causing skin and soft tissue infections both in immunocompetent and immunocompromised individuals, with more severe and disseminated infections being reported mainly in immunocompromised patients [4]. Non-tuberculosis mycobacteria (NTM) are differentiated from *Mycobacterium tuberculosis* due to a lack of human-to-human transmission [12]. They are ubiquitous in the environment, and an increasing incidence of infections has been noted since the 1950s when they were first described as human pathogens [12]. *Mycobacterium chelonae* has been associated with localized skin infections (abscesses, cellulitis), however, systemic hematogenous dissemination has also been noted [13]. There is an association between localized cutaneous and soft tissue infections with the introduction of cannula and other synthetic materials [13]. This route of exposure was potentially a source of infection for our patient as well.

Our patient initially failed two courses of oral antibiotics and was started on prednisone taper given a concern for gout in the setting of mildly elevated uric acid levels. Due to the anti-inflammatory effects of steroids, she initially responded favorably, however, this only prolonged the clinical course of the infection. A review of literature focusing on lower extremity infections caused by *Mycobacterium chelonae *showed that there is often a delay in diagnosis with median time being 7.9 months [12]. Due to persistent symptoms in the setting of an immunodeficient state, she was started on broad-spectrum antibiotics and then switched to a combination of appropriate therapy after cultures showed infection with *Mycobacterium chelonae*. These infections can be difficult to diagnose and treat, given the pathogens’ resistance to many commonly used antibiotics and the insidious onset of the infection [4]. For localized skin infections clarithromycin monotherapy can be sufficient, but given that resistance may occur if prolonged therapy is needed, treatment with two antibiotics is recommended [4].

## Conclusions

In conclusion, this case demonstrates the significance of considering patients with MGUS as immunocompromised. The differential should remain broad and allow for the consideration of atypical infections. Sub-specialists should be included early in the treatment of infection if the patient has a history of MGUS. Furthermore, we want to draw attention to the correlation between MGUS and *Mycobacterium *infection in this subset of immunocompromised patients.
